# Reversible leukoencephalopathy caused by 2 rodenticides bromadiolone and fluroacetamide: A case report and literature review

**DOI:** 10.1097/MD.0000000000025053

**Published:** 2021-03-05

**Authors:** Aili Lu, Fang Yuan, Yufei Yao, Wanxin Wen, Hongji Lu, Shibiao Wu, Lixin Wang

**Affiliations:** The Second Affiliated Hospital of Guangzhou University of Chinese Medicine, Guangzhou, China.

**Keywords:** central nervous system, coagulopathy, corpus callosum, leukoencephalopathy, rodenticide

## Abstract

**Rationale:**

With the easy access, rodenticide poisoning has been a public health problem in many countries. Characteristics of central nervous system (CNS) lesions induced by rodenticides are scarcely reported.

**Patient concerns:**

We presented a case of a 40-year-old man with seizure and consciousness disorder, coagulation dysfunction, and symmetric lesions in white matter and corpus callosum.

**Diagnosis:**

He was diagnosed with rodenticide poisoning due to bromadiolone and fluoroacetamide.

**Interventions:**

He was treated with vitamin K, hemoperfusion, acetamide, and calcium gluconate.

**Outcomes:**

His leukoencephalopathy was reversed rapidly with the improvement of clinical symptoms.

**Lessons:**

This report presented the impact of rodenticide poisoning on CNS and the dynamic changes of brain lesions, and highlighted the importance of timely targeted treatments.

## 1 Introduction

Anticoagulants are the main component of the rodenticide. The first generation of anticoagulant rodenticide was developed in 1948 and was gradually replaced in the 1970s by the second generation, namely superwarfarins, due to drug resistance.^[1,2]^ As a type of superwarfarins with high potency, bromadiolone is a common rodenticide used all over the world. Bromadiolone inhibits the carboxylation of vitamin K–dependent coagulation factors (II, VII, IX, and X) and exerts a prolonged anticoagulant effect.^[3,4]^ Fluoroacetamide is another common rodenticide which induces an accumulation of citrate and cellular metabolic disorder by blocking the tricarboxylic acid cycle.^[5]^

With the easy access, rodenticide poisoning has been a public health problem in many countries.^[6–8]^ The clinical effect of bromadiolone is associated with the exposure dosage. Most patients with bromadiolone poisoning have only minor or no effects due to the small exposure.^[7,9]^ Interfering with blood coagulation, bromadiolone induces varying degrees of hemorrhage, such as ecchymoses, gingival bleeding, epistaxis, gastrointestinal bleeding, hematuria, vaginal bleeding, and rarely SAH.^[6,10–14]^ Besides coagulopathy, patients with bromadiolone poisoning sometimes present with headache, seizure, hallucinations, dizziness, consciousness impairment and a few other symptoms of central nervous system (CNS).^[7]^ Fluoroacetamide poisoning often causes damages in heart (QT prolongation, arrhythmia, myocardial damage), digestive system (vomiting, nausea, burning sensation in the epigastrium), and CNS (seizure, aphasia, myasthenia, and coma).^[15–18]^ However, characteristics of CNS lesions induced by rodenticides are scarcely reported. Here, we presented a case with reversible leukoencephalopathy caused by bromadiolone and fluoroacetamide poisoning.

## 2 Case presentation

### 2.1 Clinical history

A 40-year-old man was referred to our neurological intensive care unit (NICU) due to unconsciousness for 1 day. He had headache 4 days ago, then he started to feel lack of energy and had generalized tonic-clonic seizure for 5 minutes. Blood tests of coagulation function in the emergency unit showed a prothrombin time of 92.6 seconds (normal: 11.0–14.5 seconds), an activated partial thromboplastin time of 52.8 seconds (normal: 28.0–45.0) and an international normalized ratio of 8.88 (normal 0.80–1.20). He received fresh frozen plasma transfusion and intravenous treatments of diazepam and valproate and in the emergency unit. This patient was unconscious on admission, so he could not provide any history of poison ingestion. His family reported no history of familial diseases and no awareness of poisoning.

### 2.2 Clinical examination and diagnosis

He had eyes opening to sound, no spontaneous motor movements, no motor movements to commands, and no speech or vocalization. His 4 limbs had normal flexion to pain stimuli. His pupil diameters are 2.0 mm. Direct and indirect light reflexes of both pupils were slow. All the brainstem reflexes were present. His muscle tone was normal, and no involuntary movements were observed. Tendon reflexes of upper limbs were normal, patellar reflexes of both legs were exaggerated. Babinski signs of both sides were positive, and meningeal irritation signs were negative. No ecchymoses or any other hemorrhage was found in his skin, conjunctiva, nose, and mouth. He had no hemoptysis, haematemesis, hematochezia, or hematuria.

**Figure 1:**
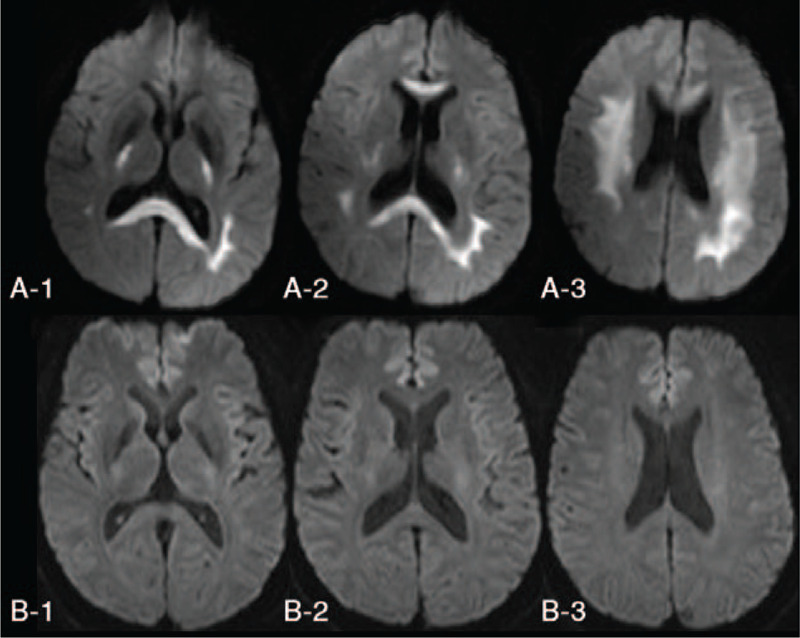
Brain Diffusion Weighted Imaging. A. On admission; B. 7 days after consciousness recovery.

His blood tests showed a mild elevation in total bilirubin (35.1 μmol/L), unconjugated bilirubin (27.2 μmol/L), and ammonia (44.0 μmol/L). Liver enzymes, creatinine, electrolytes in the blood were normal. Gastric occult blood test was positive (3+), and fecal occult blood test was negative. Brain Diffusion Weighted Imaging (DWI) showed hyperintense lesions throughout the corpus callosum and in both sides of brachium pontis, posterior limb of internal capsule, periventricular white matter, centrum semiovale, and corona radiata (Fig. 1A). Toxin testing identified bromadiolone (58 ng/ml, liquid chromatography-mass spectrometry) and fluoroacetamide (chromatography-mass spectrometry) in his blood, and fluorine (25.21 mg/g creatinine, WS/T 30-1996) in his urine. He was diagnosed with rodenticide poisoning.

### 2.3 Treatment

He received fresh frozen plasma transfusion and hemoperfusion upon his admission to NICU, together with vitamin K1 10 mg iv, pantoprazole 40 mg iv, and sodium valproate 600 mg po. As soon as the result of toxin detection came out (4 hours after NICU admission), vitamin K1 30 mg/d iv, acetamide 5 g tid im., and calcium gluconate 2 g/d iv were administered for 12 days.

### 2.4 Outcome

On the second day in NICU, he opened eyes spontaneously with accurate but slightly slurred speech and normal muscle strength in all his limbs. On the third day in NICU, his speech became clear. His coagulation function became completely normal on the fourth day in NICU, then he was transferred out of NICU. He took brain MRI examination again 7 days after consciousness recovery: previous high DWI signals in corpus callosum and in both sides of brachium pontis, posterior limb of internal capsule, PWN, centrum semiovale, and corona radiata all disappeared (Fig. 1B). He was discharged from hospital 2 weeks from the onset, and he did not take vitamin K1, acetamide, or calcium gluconate after hospital discharge. He reported no symptoms at 5-month follow-up.

## 3 Discussion

Ecchymoses and bleeding were the most common initial symptoms of rodenticide poisoning.^[6,19]^ There was even a case reporting the misdiagnosis of rodenticide poisoning as ectopic pregnancy in an 18-year-old woman.^[14]^ In this case, sudden impairment of consciousness, coagulation disorder, and symmetric lesions in periventricular white matter and corpus callosum indicated a possibility of toxic encephalopathy. Accordingly, plasma transfusion and vitamin K were given to improve coagulation function, and hemoperfusion was performed to remove toxins. As soon as the identification of toxins (bromadiolone and fluoroacetamide), vitamin K1 30 mg/d iv, acetamide 5 g tid im, and calcium gluconate 2 g/d iv were administered. His consciousness level and coagulation function were improved rapidly, and cerebral lesions were reversed.

Previous cohort studies investigated the clinical characteristics and outcomes of rodenticide poisoning. Bromadiolone and bromethalin were the most common toxicants found in rodenticide intoxication, and rodenticide exposures were mainly pediatric (under 12 years old).^[7,20]^ Accidental ingestion was the most common cause of poisoning in children, and no effects or only minor effects were usually seen due to low exposure.^[7,11]^ Intentional ingestion and unknown intake were the most frequent causes of rodenticide intoxication in adults.^[7]^ Unknown ingestion needs longer time to make a diagnosis and give targeted treatments than intentional ingestion and usually contains greater dosage than accidental intake. Therefore, rodenticide poisoning in adults due to unknown ingestion usually leads to more severe symptoms. Once the type of rodenticides was identified, targeted treatment should be administered as soon as possible.

The supplement of Vitamin K can directly ameliorate the K-dependent coagulation factor deficiency caused by long-acting anticoagulant rodenticides. So far there were no consensuses on the loading and maintenance dosage of Vitamin K. The loading dose reported by previous studies was 10 to 100 mg/d intravenously,^[21–23]^ and the maximal loading dosage was 800 mg/d orally.^[24]^ The maintenance dosage of Vitamin K reported by previous studies was quite different as well: 5 to 600 mg/d orally.^[21–24]^ A study of 56 patients with anticoagulant rodenticides poisoning showed that there was not a significant dose–effect relationship between the concentration of rodenticides and the requirement of vitamin K1 during the maintenance period.^[10]^ For severe cases, transfusion of fresh frozen plasma, prothrombin complex, and/or recombinant coagulation factor VIIa should be given. Muscle injection of acetamide is the targeted treatment for fluoroacetamide,^[16]^ and calcium therapy can ameliorate cardiac arrhythmias induced by fluoroacetamide.^[25,26]^

Several case repots presented the effects of rodenticide on CNS (Table 1). Due to the anticoagulant effect, bromadiolone can cause intracerebral hematoma.^[27]^ The brain MRI of a bromadiolone poisoning case found symmetrical patchy lesions in bilateral posterior limb of the internal capsule, splenium of corporis callosum, and bilateral centrum semiovale which were similar to the affected locations in our case.^[28]^ Tetramine and fluoroacetamide were reported to cause hypoxic–ischemic changes at hippocampal regions and cerebral cortex.^[15]^ β-fluoroethyl acetate can cause cerebellar atrophy,^[29]^ and bromethalin may lead to leukoencephalopathy.^[7]^ In this case, we found that leukoencephalopathy was reversed with the improvement of clinical symptoms.

**Table 1:** Reported neuroimaging findings associated with rodenticides.

Case	Rodenticides	Neuroimaging findings	Treatment	Outcome
Zuo et al, 2019^[27]^	bromadiolone	CT: intracerebral haematoma	In-hospital: vitamin K (30 mg q8h) + fresh frozen plasma (800 ml in total)After hospital discharge: Vitamin K (30 mg, q8 h) for 6 months.	No obvious hemorrhage in brain
Wang et al, 2017^[28]^	bromadiolone	MRI: symmetrical patchy lesions in bilateral posterior limb of the internal capsule, splenium of corporis callosum, and bilateral centrum semiovale.	Vitamin K and blood plasma (unknown dosage)	Relief from confusion and dysphoria
Wang et al, 2016^[15]^	tetramine +fluoroacetamide	CT: hypoxic–ischemic changes lightly at hippocampal regions and cerebral cortex	Case 1: no treatment.Case 2: unknown.	Case 1: Death.Case 2: Recovered.
Jin et al, 2017^[29]^	β-fluoroethyl acetate	MRI: cerebellar atrophy	Unknown	Unknown
Feldman et al, 2019^[7]^	bromethalin	MRI: leukoencephalopathy (non-specified)	Unknown	Pediatric: 96.38% had no effects, 3.32% had minor effects, and 0.45% had moderate effects.Patients >12 yrs: 65.73% had no effect, 25.58% had minor effects, 5.88% had moderate effects, 2.30% had major effects, and 0.51% died.

## 4 Conclusions

We reported a case of rodenticide poisoning presented with seizure, consciousness disorder, and coagulation dysfunction. His brain DWI showed symmetric lesions in white matter and corpus callosum. After receiving vitamin K, hemoperfusion, acetamide, and calcium gluconate, he restored consciousness and his leukoencephalopathy was rapidly reversed. This report presented the impact of rodenticide poisoning on CNS and the dynamic changes of brain lesions, and highlighted the importance of timely targeted treatments.

## Author contributions

**Conceptualization:** Aili Lu, Fang Yuan.

**Data curation:** Yufei Yao, Wanxin Wen, Hongji Lu, Shibiao Wu.

**Formal analysis:** Aili Lu, Fang Yuan.

**Investigation:** Yufei Yao, Wanxin Wen, Hongji Lu, Shibiao Wu.

**Supervision:** Lixin Wang.

**Writing – original draft:** Aili Lu, Fang Yuan, Yufei Yao.

**Writing – review & editing:** Lixin Wang.
