# Reversible leukoencephalopathy caused by 2 rodenticides bromadiolone and fluoroacetate: A case report and literature review: Erratum

**DOI:** 10.1097/MD.0000000000026668

**Published:** 2021-07-16

In the article, “Reversible leukoencephalopathy caused by 2 rodenticides bromadiolone and fluoroacetate: A case report and literature review” which appears in Volume 100, issue 9 of *Medicine*, the word fluoroacetate was spelled incorrectly in the title and should have been written as fluroacetamide. The title has been updated to “Reversible leukoencephalopathy caused by 2 rodenticides bromadiolone and fluroacetamide: A case report and literature review”.
